# Sharpening of expression domains induced by transcription and microRNA regulation within a spatio-temporal model of mid-hindbrain boundary formation

**DOI:** 10.1186/1752-0509-7-48

**Published:** 2013-06-25

**Authors:** Sabrina Hock, Yen-Kar Ng, Jan Hasenauer, Dominik Wittmann, Dominik Lutter, Dietrich Trümbach, Wolfgang Wurst, Nilima Prakash, Fabian J Theis

**Affiliations:** 1 Institute of computational Biology, Helmholtz Center Munich, Ingolstädter Landstr. 1, Neuherberg 85764, Germany; 2 Department of Mathematics, University of Technology Munich, Boltzmannstr. 3, Garching 85747, Germany; 3 Institute of Developmental Genetics, Helmholtz Center Munich, Ingolstädter Landstr. 1, Neuherberg 85764, Germany; 4 Deutsches Zentrum für Neurodegenerative Erkrankungen (DZNE) Standort München, , Schillerstr. 44, Munich 80336, Germany; 5 Max-Planck-Institute of Psychiatry, Kraepelinstr. 2, Munich 80804, Germany

**Keywords:** Mid-Hindbrain Boundary, miRNA Modeling, Spatio-Temporal Model, Developemental Biology

## Abstract

**Background:**

The establishment of the mid-hindbrain region in vertebrates is mediated by the isthmic organizer, an embryonic secondary organizer characterized by a well-defined pattern of locally restricted gene expression domains with sharply delimited boundaries. While the function of the isthmic organizer at the mid-hindbrain boundary has been subject to extensive experimental studies, it remains unclear how this well-defined spatial gene expression pattern, which is essential for proper isthmic organizer function, is established during vertebrate development. Because the secreted Wnt1 protein plays a prominent role in isthmic organizer function, we focused in particular on the refinement of *Wnt1* gene expression in this context.

**Results:**

We analyzed the dynamics of the corresponding murine gene regulatory network and the related, diffusive signaling proteins using a macroscopic model for the biological *two-scale signaling process*. Despite the discontinuity arising from the sharp gene expression domain boundaries, we proved the existence of unique, positive solutions for the partial differential equation system. This enabled the numerically and analytically analysis of the formation and stability of the expression pattern. Notably, the calculated expression domain of *Wnt1* has no sharp boundary in contrast to experimental evidence. We subsequently propose a post-transcriptional regulatory mechanism for *Wnt1* miRNAs which yields the observed sharp expression domain boundaries. We established a list of candidate miRNAs and confirmed their expression pattern by radioactive in situ hybridization. The miRNA *miR-709* was identified as a potential regulator of *Wnt1* mRNA, which was validated by luciferase sensor assays.

**Conclusion:**

In summary, our theoretical analysis of the gene expression pattern induction at the mid-hindbrain boundary revealed the need to extend the model by an additional *Wnt1* regulation. The developed macroscopic model of a two-scale process facilitate the stringent analysis of other morphogen-based patterning processes.

## Background

Patterning phenomena based on the activation of target genes in a concentration-dependent manner by diffusive signaling molecules, so called morphogens, are of great biological importance [1,2] as shown, e.g., in Drosophila [3] and mice [4]. Model-based approaches are often used to investigate the formation of morphogen gradients and to analyze the sufficiency of the known regulatory mechanisms. Such models do not consider the cells individually but rather as a continuum and describe the macroscopic (or homogenized) dynamics of the process. The emerging macroscopic models are theoretically justified and have already been employed in various biological contexts (see, e.g., [5,6]). An interesting process of such type is the patterning of the neural plate, a precursor tissue, which gives rise to the vertebrate central nervous system (CNS).

Shortly after gastrulation, the neural plate undergoes patterning along the anteroposterior axis into four distinct regions. These regions are the presumptive forebrain, midbrain, hindbrain and spinal cord. This subdivision relies on a well-defined and locally restricted expression of genes mediating the action of short and long-range signaling centers, so-called secondary organizers [7]. Of special interest is the isthmic organizer (IsO), the secondary organizer located at the boundary between the prospective mid- and hindbrain. The IsO is necessary and sufficient for the development of the mid-hindbrain region (MHR) [8] and it is characterized by the localized expression of several transcription and diffusive signaling molecules.

In the context of the IsO, eight genes are of special interest: *orthodenticle homologue 2* (*Otx2*) and *gastrulation brain homeobox 2* (*Gbx2*), two transcription factors initially expressed in the anterior and posterior, respectively, part of the developing embryo abutting each other, and whose expression boundary determines the position of the future mid-hindbrain boundary (MHB); *fibroblast growth factor 8* (*Fgf8*), which is necessary for the patterning of the MHR, and *wingless-type MMTV integration site family member 1* (*Wnt1*), required for the maintenance of the IsO, two “morphogens” secreted from the posterior and anterior, respectively, border of the MHB; and the *Engrailed* (*En1* and *En2*) as well as the *paired box* (*Pax2* and *Pax5*) transcription factors acting down- or upstream of Fgf8 and Wnt1 and mediating their patterning/maintenance function at the MHB [8]. *En1* and *En2* are subsumed under the identifier *En*, and *Pax2* and *Pax5* are subsumed under the identifier *Pax* due to their conserved biological function in mid-/hindbrain boundary (MHB) development. All of these genes start to be expressed around mouse embryonic day (E) 8.5 of development. Various loss- and gain-of-function experiments demonstrated that at E10.5, these genes are interdependent and their expression patterns are maintained at least until E12.5. These genes build the basis of a gene regulatory network (GRN) that is necessary for the sharpening and subsequent maintenance of the specific IsO gene expression pattern [8]. One crucial aspect of the IsO function is the spatio-temporally defined and localized expression of its constituent genes, including the formation of sharp boundaries between the gene expression domains (for a comprehensive review see [8]).

**Figure 1:**
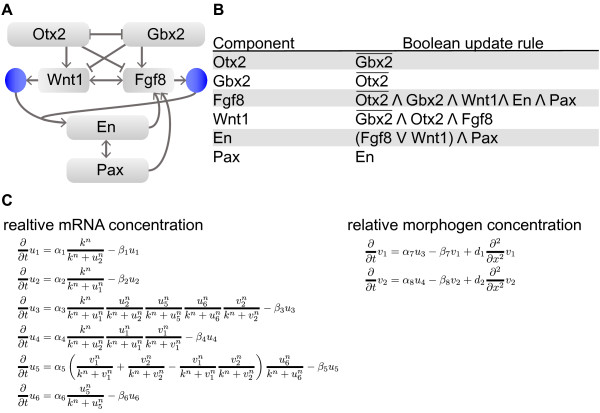
**MHB gene regulatory network and model.(A)** Gene regulatory network of the six IsO genes considered (adapted from [10]). The two morphogens, which are encoded by *Fgf8* and *Wnt1* are shown as blue circles. Arrows represent activation/production and bars represent inhibition. **(B)** Boolean update functions for each node [10]. ∧ is the Boolean AND, ∨ is the Boolean OR. **(C)** Partial differential equation model derived from the Boolean update functions [10,12,13]. State variables of the macroscopic model of the two-scale process are: *u*(*t*,*x*) = (*Otx2*(*t*,*x*),*Gbx2*(*t*,*x*),*Fgf8*(*t*,*x*),*Wnt1*(*t*,*x*),*En*(*t*,*x*),*Pax*(*t*,*x*))^T^ (cell-specific) and *v*(*t*,*x*) = (Fgf8(*t*,*x*),Wnt1(*t*,*x*))^T^ (diffusive).

Experimental data obtained from in situ hybridization experiments have been employed to determine the expression domains/patterns of the IsO genes (see [9] for a detailed description of *in situ* hybridization methods). These data are semi-quantitative and capture the level of transcription relative to the minimal and maximal transcription in the same tissue. Based on those experiments the GRN schematically depicted in Figure 1A was inferred [10]. Using artificial thresholds Wittmann et al. [10] constructed the Boolean model shown in Figure 1B. This Boolean model has been shown to be able to generate and robustly maintain the experimentally observed steady state pattern [10-12]. However, the usage of artificial thresholds results in the loss of information. To exploit all information contained in the data, Wittmann et al. [10] derived a continuous spatio-temporal model using a discrete to continuous transformation of the Boolean network. Therefore, the discrete Boolean update functions are replaced by Hill-type functions [10,13]. The resulting macroscopic model describes the time evolution of transcription factor activities. As these activities are confined to individual cells there is no spatial evolution on this level. Hence the equations can be treated as ordinary differential equations (ODEs) for a each point in space. The tissue scale morphogen gradients and their dynamics are described using diffusion equations. Both models are coupled to account for secretion and uptake of morphogens, the interface between the models, and the full system is shown in Figure 1C. Using this semi-quantitative spatial modeling approach, several interesting aspects of the MHB formation can be assessed, which cannot be studied using Boolean models [10].

The class of macroscopic models for two-scale processes provides the basis of the following theoretical and numerical analysis of IsO gene network and signaling proteins. Complementing previous work we address the existence, uniqueness, positivity of solutions for the model. We extend previous studies and analyzed the induction of the gene expression patterns at the MHB, especially, we focused on the formation of sharp spatial patterns. Therefore, we introduced theoretical and numerical tools for semi-quantitative two-scale processes. Using these tools we found that the model has to be extended by a regulation of *Wnt1* expression to describe the sharp expression pattern observed *in vivo*. Based on this insight we analyzed, which regulation mechanisms allows for the specific *Wnt1* expression pattern, focusing on post-transcriptional regulation by miRNAs. MiRNAs are short (∼ 22 nucleotides long) non-coding RNAs which post-transcriptionally regulate the gene (mRNA) expression [14-16]. This is achieved by binding of the miRNA to the mRNA, repressing the translation of the mRNA into protein. Furthermore, if the degree of miRNA-mRNA complementarity is high miRNAs induce the decay of the mRNA [17-19]. MiRNAs play a critical role in diseases such as cancer [20] and neuro-degeneration [21] as well as embryonic development [16,22,23].

## Results

### Macroscopic, semi-quantitative model of a two-scale process

**Figure 2:**
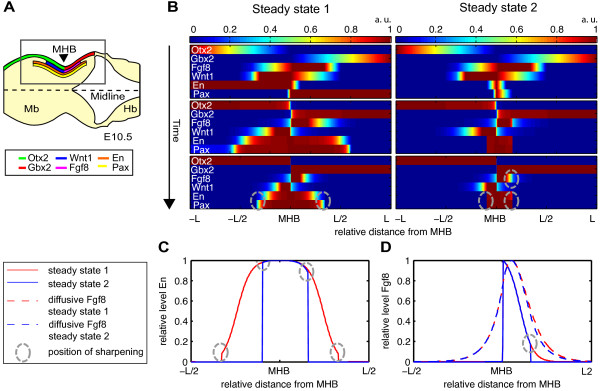
**Dynamics and steady states of the MHB model.(A)** Top view of steady state expression pattern of the six IsO genes *Otx2, Gbx2, Wnt1, Fgf8, En* (subsuming *En1* and *En2*) and *Pax* (subsuming *Pax2* and *Pax5*) in the MHR around the MHB (black arrow head) of the mouse embryo at E10.5. The midline represents the anterior-posterior axis of the embryo. **(B)** Dynamics of the approximated steady states for the parameters *α*_*i*_ = 1 and *β*_*i*_ = 1 for *i* = 1,…,8, all Hill interaction parameters are identical with *n* = 2 and *k* = 0.1. Here the sum of gene and morphogen, i.e. *Fgf8* plus Fgf8 and *Wnt1* plus Wnt1, is plotted for better visibility. Both steady states show a sharpening of the *En* and *Pax* domain marked by the dashed circles. The second steady state shows a sharp *Fgf8* boundary indicated by the dashed circle. Both simulations were run for *T* = 0 to *T* = 50, shown here are *T* = 0 (first panel), *T* = 1 (second panel) and *T* = 50 (third panel) for a grid of size *N* = 1,000. At *T* = 50 we found that the system has reached a steady state. **(C)** Enlarged view of the sharpening, i.e. discontinuities, in the *En* expression level arising in both steady state. **(D)** Enlarged view of the sharpening, i.e. discontinuities, in the *Fgf8* expression level arising only in the second steady state. Abbreviations: MHB: mid-hindbrain boundary, Mb: midbrain, Hb: hindbrain.

The macroscopic spatio-temporal model used to describe the patterning process during the MHB formation considers two biological scales. The single cell scale, on which a semi-quantitative model for the transcription factor activation is employed, and the tissue scale, on which morphogen concentrations and gradients are regarded. The state of the individual cells is described by the activities of the four transcription factors, *Otx2*, *Gbx2*, *En* and *Pax* and the two morphogen encoding genes *Fgf8*, *Wnt1*, which are described in the Background section, and the morphogens Fgf8 and Wnt1. The interactions are schematically displayed in Figure 1A. The joint dynamics of transcription factor activities *u*(*t*,*x*) = (*u*_1_(*t*,*x*),…,*u*_6_(*t*,*x*))^T^ and morphogen *v*(*t*,*x*) = (*v*_1_(*t*,*x*),*v*_2_(*t*,*x*))^T^ are described by an eight-dimensional system of partial differential equations (PDEs) on the spatial domain *x* ∈ *U* = [ − *L*,*L*]. As the up- and down-regulation of transcription depends only on the concentration of transcription factors within the individual cells and the local concentration of morphogens, the time evolution of the transcription factor activity is governed by a reaction equation,

$$\begin{array}{cc} \frac{\partial }{\mathrm{\partial t}}u_{i}(t,x)=\alpha_{u_{i}}B_{i}(x,u(t,x),v(t,x))-\beta_{u_{i}}u_{i}(t,x),\; & \\ \;i=1,\ldots ,6,\; & \end{array}$$

with production term $\alpha_{u_{i}}B_{i}(x,u(t,x),v(t,x))$ and degradation rate $\beta_{u_{i}}$. While $\alpha_{u_{i}}$ and $\beta_{u_{i}}$ are constant, *B*_*i*_(*x*,*u* (*t*,*x*),*v*(*t*,*x*)) is potentially a nonlinear function of *x*, *u*(*t*,*x*) and *v*(*t*,*x*). *B*_*i*_ represents the Hill-type regulation of transcription factor *i* by transcription factors and morphogens (Figure 1C). The initial conditions for the transcription factor activity are given by the spatial functions *u*_0*i*_(*x*), *i* = 1,…,6, which describe quantitatively the expression pattern at E8.5 (Figure 2B upper panels). Hence,

(1) $$u_{i}(0,x)=u_{0i}(x),\;i=1,\ldots ,6.$$

We assumed that the morphogens are produced proportionally to the activation of the corresponding transcription factor *u*_*i*_ which resembles a constant translation of mRNA to protein. The dynamics of the morphogen concentration is then governed by a reaction-diffusion equation,

$$\begin{array}{cc} \frac{\partial }{\mathrm{\partial t}}v_{j}(t,x)=\alpha_{v_{j}}u_{i}(x,t)-\beta_{v_{j}}v_{j}(t,x)+d_{j}\frac{\partial^{2}}{\partial x^{2}}v_{j}(t,x),\; & \\ \;j=1,2,\; & \end{array}$$

in which $\alpha_{v_{j}}$, $\beta_{v_{j}}$, and *d*_*j*_, denote the constant synthesis, degradation, and diffusion rate, respectively. In the following, we consider the anterior-posterior direction of the neural tube, which is large in comparison to the MHR and the expected diffusion length scale. Hence, no morphogen arrives at the border, i.e. we have zero morphogen concentration, *v*_*j*_(− *L*,*t*) = *v*_*j*_(*L*,*t*) = 0. The initial conditions are given by a two-dimensional vector of spatial functions *v*_*j*0_(*x*), *j* = 1,2, corresponding to the pattern at E8.5. Hence,

$$v_{j}(0,x)=v_{0j}(x),\;j=1,2.$$

As the PDEs for *v*(*t*,*x*) are linearly coupled with the ODEs for *u*(*t*,*x*), we can conclude that solutions exist for all time points *t* ∈ [0,*∞*) [24]. Furthermore, the solution is unique, positive and in $C([0,T];L^{2}(U;R^{8}))$ if the initial condition vector (*u*_0_,*v*_0_)^T^ is positive and bounded [24]. This means that we obtain a solution which is continuous with respect to time and a square integrable function with respect to space and we can consider the asymptotic steady state of the system. Furthermore, the insight that the solutions are square integrable in *x* ensures the convergence of finite-difference methods, which are described in “Methods”. In the simulation, special attention has to be paid to the non-differentiabilities occuring at least for *Otx2* and *Gbx2* in the stationary limit [12].

### MHB model predicts stable steady state patterns

Biologically, one important characteristic of the GRN at the IsO is the activation of the genes in a precise spatio-temporal manner and the correct positioning of their expression domains [8]. Once the pattern is reached it has to be maintained even if the whole system is slightly perturbed for example by transcription noise. This implies that the model has to exhibit a stable stationary limit solution, which resembles the steady state gene expression pattern that is observed at E10.5.

Due to the nonlinear coupling, the analytical calculation of the steady state is infeasible for most components of the system. Only for *Otx2* and *Gbx2* an analytical calculation of the steady state solution is possible because they form a simple genetic toggle-switch system independent of the other components. This system is well studied and we knew that it exhibits separated expression domains depending on the interaction strength of the two players [25,26]. For simplicity and because we lacked detailed knowledge of the interaction parameters of *Otx2* and *Gbx2*, we assumed a symmetric parameter setup, i.e.

$$\begin{array}{lll} \frac{\partial }{\mathrm{\partial t}}u_{1}(t,x) & =\frac{\alpha_{1}k^{n}}{k^{n}+u_{2}^{n}}-\beta_{1}u_{1}(t,x)\; & \;\\ \frac{\partial }{\mathrm{\partial t}}u_{2}(t,x) & =\frac{\alpha_{2}k^{n}}{k^{n}+u_{1}^{n}}-\beta_{2}u_{2}(t,x),\; & \; \end{array}$$

with *α*_1_ = *α*_2_ and *β*_1_ = *β*_2_. Here *u*_1_(*t*,*x*) denotes the expression of *Otx2* and *u*_2_(*t*,*x*) denotes the expression of *Gbx2*. Based on the analytical calculation of the steady state we found that the parameter values for which we obtained a steady state with separated expression domains have to satisfy $\frac{\beta_{1}}{\alpha_{1}}>{\left(n-1\right)}^{1+1/n}/n$. Furthermore, the point *x*^∗^ where both expression domains abut each other in the stationary limit is determined by the initial conditions of *Otx2*, denoted by *u*_01_(*x*), and *Gbx2*, denoted by *u*_02_(*x*). If *u*_01_(*x*)>*u*_02_(*x*) for *x*<*x*^∗^ and *u*_01_(*x*)<*u*_02_(*x*) for *x*>*x*^∗^ then the boundary is placed at *x*^∗^.

In the following, the MHB is placed in the middle of the considered interval, *x*^∗^ = 0. Furthermore, similar to [10,12], we set *n* = 2, *k* = 0.1, and *α*_*i*_ = *β*_*i*_ = 1 for *i* = 1,…,8. The diffusion coefficients of *Fgf8* and *Wnt1* were reduced compared to previous publications to *d*_1_ = *d*_2_ = 0.001. For the chosen diffusion parameters the values $v_{1}(t,-\frac{L}{2})$ and $v_{2}(t,\frac{L}{2})$ are approximately zero, ensuring that the Dirichlet boundary conditions have no significant influence on the dynamics or the steady state of the systems, as assumed in the modeling process. For this setup we performed an extensive simulation study to determine the non-trivial steady states of the system (see “Methods” for details). If the initial transcription factor and morphogen patters are unimodal, we find two stable, non-zero stationary limit solutions. While the steady state 1 (Figure 2B, left panel) shows a maximal width for the expression domain of *En* and *Pax*, for steady state 2 (Figure 2B, right panel) this width is minimal. The panels in Figure 2B show exemplary trajectories leading to the steady states. The simulation study indicates, that these are the only steady states reachable from initially unimodal expression domains.

For initially multimodal expression domains, we observed more complex expression pattern, e.g., single spots of *En* or *Pax* expression. These arise as *En* or *Pax* locally exceed the threshold of their mutual activation, yielding isolated point regions with maximal *En* or *Pax* expression, respectively. The spots are positioned in the region between the minimal and maximal width expression domain given by the steady states mentioned above. We did not consider the steady states with expression spots as they are not thought to be of biological relevance, however this shows that the model is capable of producing a whole spectrum of steady states. Subsequently, we analyzed the stability of the two non-trivial steady states to understand the temporal dynamics in their close proximity. We found that both non-trivial steady states are stable. For both steady states that we consider, the expression of *Otx2*, *Gbx2* and *Wnt1* are the same, whereas for *Fgf8*, *En* and *Pax* we observe a difference in the width of the individual expression domains.

### Predicted steady states coincide qualitatively with the experimental observations

We compared our steady state solutions and their dependence on initial conditions and parameters to experimentally validated expression patterns. In our model the only factor which determines the position of the MHB is the initial expression pattern of *Otx2* and *Gbx2*. This major role of *Otx2* and *Gbx2* has already been shown in *in vivo* knock-out or knock-in experiments, in which a change of *Gbx2* and *Otx2* expression domains also led to a change in the position of the MHB [27,28]. Furthermore, both steady states show a restriction of the *En* and *Pax* expression to the MHB region, which can also be observed *in vivo*[8]. The steady state with the more restricted initial pattern (Figure 2B right panels and Figure 2C) shows a tighter expression domain. This tighter domain is due to the activating interaction between *En* and *Pax*, whereas the other steady state is dominated by the activating interaction of *Fgf8* and *Wnt1* with *En*.

Concerning the expression domain of *Fgf8* we found that in steady state 2 the expression is restricted in posterior direction due to the regulation of *Fgf8* by *En* and *Pax*. Only in this steady state a sharp boundary of the *Fgf8* expression domain in posterior direction occurs (indicated by the dashed circles in Figure 2B,D). This agrees with the experimental observations that the expression of *Fgf8* is restricted to a ring at the caudal border of the MHB [29]. Furthermore, we concluded that the interactions which are not necessary to maintain the expression pattern according to [10], are required to sharpen the expression domain of the morphogen *Fgf8*. Those interactions are the activation of *En* by *Fgf8* and *Wnt1* and the activation of *Fgf8* by *En* and *Pax*, which were not considered in [12].

Finally, we considered the expression pattern of *Wnt1*, which is the same in both steady states. The IsO is a signaling center and one of its main tasks is to generate a well-defined *Wnt1* gradient. The gradient results from the sharply restricted and positioned expression domain of *Wnt1*. However, unfavorable smooth interfaces of expression domains occur if expression is only regulated by a morphogen in our case *Fgf8*[30]. Depending on the diffusion coefficient of *Fgf8* and the activation of *Wnt1* with *Fgf8*, the expression domain of *Wnt1* becomes increasingly smooth. This disagreement with the experimental results where *Wnt1* is expressed in a narrow ring at the rostral border of the MHB with a clearly visible boundary [8,31-33]. For a detailed illustration and semi-quantitative assessment of the expression domain of *Wnt1* we refer to the EMAP eMouse Atlas Project [34] (http://www.emouseatlas.org/).

In the following section we will discuss possible post-transcriptional sharpening mechanisms for the *Wnt1* expression profile. As the more restricted *Fgf8* expression pattern in steady state 2 is closer to the experimental observations [29], we will use this steady state in the following analysis, however, the results are similar if steady state 1 is used. In particular the *Wnt1* expression patterns are alike for both steady states.

### Sharpening of the *Wnt1* expression by miRNA interactions

As our simulations showed no sharply delimited expression domain in the anterior direction for the *Wnt1* expression domain (see Figure 2B), there has to exist an unknown mechanism enforcing the sharpening of the *Wnt1* profile over time, which is not considered in the model. In this work we consider post-transcriptional miRNA regulation as recent studies proved that miRNAs regulation is particular active at low mRNA copy numbers, which occur in our model simulation at the *Wnt1* expression domain boundary, and can induce gene expression thresholds [30,35]. This might results in a spatial sharpening of the target expression domain in the overlapping region [30,35] via one of the following mechanisms: 

i) The miRNA binds transiently to the mRNA and only the mRNA is degraded (low degree of complementarity).

ii) The mRNA-miRNA complex is degraded (high degree of complementarity).

iii) The mRNA-miRNA complex is degraded and unbound miRNAs are actively transported between neighboring cells [36,37].

**Figure 3:**
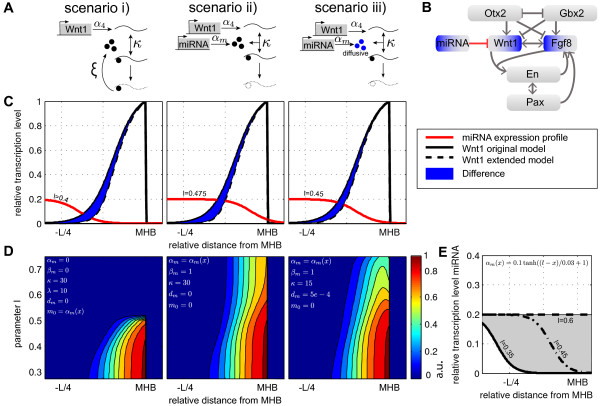
**Extension of the MHB signaling model by miRNA regulation.(A)** Illustration of the three considered miRNA regulation scenarios. Boxes describe the encoding gene, black dots represent the mature miRNA and the curved lines represent the transcribed mRNA. The parameters *α*_*m*_, *α*_4_, *κ* and *ξ* are the parameters considered in the model. **(B)** Graph of extended regulatory network. **(C)** Sharpening of the *Wnt1* expression domain for a given miRNA expression profile parameters for the simulations given in **(D)**. Each plot corresponds to the scenario above it. **(D)***Wnt1* expression level depending on miRNA expression profile. The expression profile is varied by the parameter *l*, which determines the size of the overlay with the *Wnt1* expression domain. For all simulations we used the same parameters for the original MHB model as in Figure 2B. **(E)** MiRNA expression level and its dependence on the parameter *l*.

The scenarios are illustrated in Figure 3A. It is also observed that miRNAs do not lead to a degradation but to a translational inhibition, which would results in a complex accumulation and a low degradation rate in scenario ii) and iii). Mathematically, the model extensions are given by

$$\begin{array}{cc} \frac{\partial }{\mathrm{\partial t}}u_{4}(t,x)=\alpha_{u_{4}}B_{4}(x,u(t,x),v(t,x))-\beta_{u_{4}}u_{4}(t,x)\; & \\ \;-\mathrm{\kappa m}(t,x)u_{4}(t,x)\; & \\ \frac{\partial }{\mathrm{\partial t}}m(t,x)=\alpha_{m}(x)-\beta_{m}m(t,x)-\mathrm{\kappa m}(t,x)u_{4}(t,x)\; & \\ \;+\mathrm{\lambda \xi c}(t,x)+d_{m}\frac{\partial^{2}}{\partial x^{2}}m(t,x)\; & \\ \frac{\partial }{\mathrm{\partial t}}c(t,x)=\mathrm{\kappa m}(t,x)u_{4}(t,x)-\mathrm{\lambda c}(t,x)\; & \end{array}$$

with boundary conditions

$$\begin{array}{cc} m(t,-L)=\alpha_{m}(-L)\;\text{and}\;m(t,L)=\alpha_{m}(L)\; & \\ c(t,-L)=0\;\text{and}\;c(t,L)=0\; & \end{array}$$

and initial condition

$$m(0,x)=m_{0}(x)\;\text{and}\;c(0,x)\equiv 0.$$

Here, *m*(*t*,*x*) is the relative concentration of miRNA, *c*(*t*,*x*) is the relative concentration of mRNA-miRNA complex, and *u*_4_ is the relative *Wnt1* mRNA level. *β*_*m*_ is the degradation rate, *d*_*m*_ is the diffusion coefficient of the miRNA and *α*_*m*_(*x*) is the space dependent production profile. Following the suggestions in [30] we used a sigmoidal shaped function *α*_*m*_(*x*) = *p*_1_(tanh((*l* − *x*)/*p*_2_) + *p*_4_) to model the spatial dependence of the miRNA synthesis. The interaction strength of miRNA and *Wnt1* mRNA is given by the binding rate *κ*, the degradation rate of the resulting mRNA-miRNA complex is *λ*, and the degree of miRNA recycling is denote by *ξ*.

Given this general model, the three scenario can be described by different parameter sets. For scenario i), the turn-over parameters of the miRNA are set to zero *α*_*m*_(*x*)≡0 and *β*_*m*_ = 0, assuming time-independent overall miRNA level. Furthermore, miRNA is assumed to be completely recycled, *ξ* = 1, but not transported, *d*_*m*_ = 0. In contrast, for scenario ii) and iii) *ξ* = 0, *α*_*m*_(*x*)≥0 ∀*x* ∈ *U*>0, and *β*_*m*_>0. For these to parameters merely the diffusion coefficient is different, i.e. for ii) *d*_*m*_ = 0 and for iii) *d*_*m*_>0. For all scenarios *κ*>0 and *λ*>0.

For this extended macroscopic model the existence, positivity and uniqueness is also guaranteed if and only if *α*_*m*_(*x*) is a bounded, Lipschitz continuous function. This is the case as we assumed *α*_*m*_(*x*) to be a production profile with values in [0,1]. Hence, we could conclude that unique, positive solutions exist for the extended model and we can simulate the model with the proposed algorithm. It is not surprising that an overlap of the miRNA and *Wnt1* profile leads to a sharpening of the *Wnt1* expression profile. However, we are especially interested in the spatial shape an miRNA expression domain must have to sharpen the *Wnt1* boundary sufficiently as this can easily be compared to experimental findings and will lead to predictions for possible regulating miRNAs. In this context we defined a sharpening as reduction of *Wnt1* in anterior direction and no reduction at the MHB, i.e. an increase in the second derivative with respect to *x* of the *Wnt1* transcription level for *x* ∈ (0,*L*/2).

We simulated the three scenarios for different parameter sets (*α*_*m*_,*d*_*m*_,*κ*,*λ*) using the same initial conditions and parameters for the original model components and the profile function *α*_*m*_(*x*) = 0.1(tanh((*l* − *x*)/0.3) + 1) with varying *l* (the profile is shown in Figure 3E). A representative simulation result is shown in Figure 3C). In addition, we studied the influence of the miRNA expression domain on the sharpening effect in the different scenarios. Therefore we varied the overlap of the domains by increasing the profile function parameter *l* from 0 (no-overlap) to 1 (constant production along the whole MHR). The results are shown in Figure 3D. We found that for scenario i) and ii) the miRNA level and the *Wnt1* expression domain have to overlap in the region where *Wnt1* shows intermediate expression, i.e. *l* ∈ [0.4,0.5]. For both scenarios a complete overlap of both domains leads to a overall reduction of *Wnt1* and we see no specific sharpening, especially for scenario i) a complete overlap led to a *Wnt1* knock-down state for the set of considered parameters. For scenario iii) the domains must only slightly overlap due to diffusion, if the domains strongly overlap the miRNA diffusing from the posterior direction leads to a blurring of the *Wnt1* domain at the MHB. We conclude that this scenario is not suitable for the MHB model if we have a strongly overlapping miRNA domain. In the following, we focus on scenario i) for a low and ii) for a high degree of mRNA-miRNA complementarity [17-19].

### miR-709 regulates *Wnt1* mRNA expression in vitro

Up to this point, hypotheses about a possible regulation of *Wnt1* by miRNAs was based on available knowledge about miRNA-mRNA interaction [30,35] and simulation studies of the MHB model. To gain additional insight, a search for experimental evidence of miRNAs possibly regulating the *Wnt1* mRNA expression was conducted. Therefore, we performed a miRNA target prediction and experimentally validated the predicted miRNAs by determining their expression pattern in the developing mouse embryo (with a special focus on the MHR) and their ability to target the *Wnt1* mRNA (3’UTR) *in vitro*.

We identified potential miRNAs targeting the *Wnt1* mRNA using a combination of several target prediction tools (see “Methods”). To reduce the number of false-positive predictions, we post-processed the resulting list using logic filtering. Therefore, we defined a logical state (ON/OFF) table for the three mouse embryonic stages, namely E8.5, E10.5 and E12.5, including *Wnt1* and the other five IsO genes. E12.5 is considered as the IsO gene expression pattern, which has refined to its sharp domains at E10.5, is still maintained at E12.5. Assuming that a functional miRNA targeting *Wnt1* changes the amount of produced Wnt1 protein and hence the expression state of genes downstream of Wnt1, we concluded that those genes change its expression state during miRNA expression. The target prediction was filtered for those miRNAs that target at minimum two “OFF genes” and no “ON genes” for each defined developmental stage. This resulted in a list of four miRNAs possibly regulating *Wnt1* mRNA expression (Additional file 1: Figure S1). From these possible candidate miRNAs (*miR-705* and *miR-709*) were selected by ranking the interactions according to the prediction scores provided by the distinct prediction tools.

To establish whether these two predicted miRNAs are expressed at the MHB in a pattern that is consistent with the model assumptions, their transcriptional profile in E10.5 and E12.5 wild-type mouse embryos was determined. Therefore, we used a very sensitive radioactive *in situ* hybridization method (for details see “Methods”) to detect the expression profile of these miRNAs along the entire anterior-posterior extent of the MHR on sagittal sections of these embryos.

**Figure 4:**
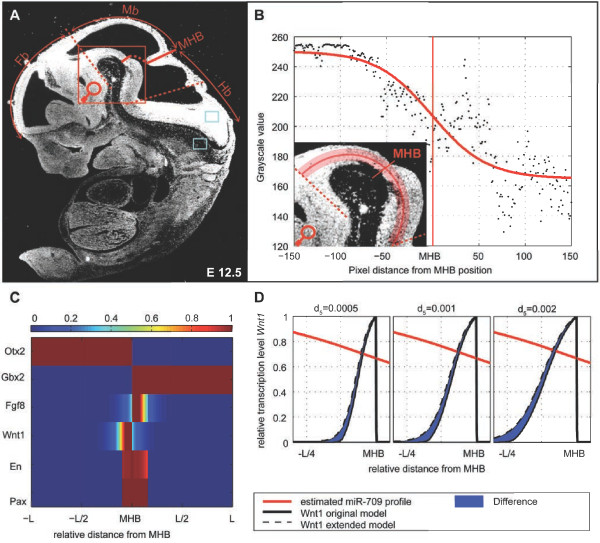
**Analysis of the*miR-709* expression in the MHR close to the MHB of the developing mouse embryo at E12.**5. **(A)** Representative sagittal section through an E12.5 CD-1 mouse embryo, hybridized with a radioactive *mmu-miR-709* LNA oligonucleotide probe. At E12.5, *miR-709* is strongly expressed in the ventral forebrain and midbrain and declines towards the ventral rostral hindbrain. **(B)** Shows the high magnification of the ventral MHR region (delimited by the dashed red lines in A) and the 300 pixel long and 15 pixel wide region along which we analyzed the profile of *miR-709*. The plot shows the grayscale values at each point along the region, calculated using the software ImageJ, and the expression profile *α*_*m*_(*x*) estimated using least-squares optimization. **(C)** Simulation of the extended MHB model for scenario ii). We choose the same parameter set used in Figure 2B right panels and *β*_*m*_ = 0.1, *d*_*m*_ = 0 and *κ* = 20. For the simulation, the miRNA grayscale profile was normalized using the mean grayscale values of the light blue boxes in A. **(D)** Comparison of the *Wnt1* expression profile in the original and extended model for three diffusion coefficients for *Fgf8* (*d*_5_). Abbreviations: FB, forebrain; Mb, midbrain; MHB, mid-hindbrain boundary; Hb, hindbrain.

In the E10.5 mouse embryo, *miR-705* is expressed only very weakly across the MHB and in the midbrain and rostralmost hindbrain, whereas *miR-709* is expressed strongly and uniformly across the MHB and in the midbrain and rostral hindbrain (see Additional file 2: Figure S2 and Additional file 2: Figure S3). Such a profile would lead to an overall reduction of *Wnt1* mRNA in the model proposed but has no sharpening effect. In the E12.5 mouse embryo, by contrast, *miR-705* was expressed strongly and uniformly in the entire MHR, including the midbrain, MHB and rostral hindbrain. However, we noted a graded expression of *miR-709* across the MHB in the ventral MHR (which is the region used to determine the expression profiles of the other IsO genes in the considered model) at this stage. Transcription of *miR-709* at E12.5 was highest in the midbrain, declined towards the MHB and was lowest in the rostral hindbrain, the region of the hindbrain that is under the influence of the IsO (see Figure 4A). This graded *miR-709* expression profile became even more evident when we calculated the grayscale profile of *miR-709* expression across the MHR, i.e. from the anterior end of the midbrain to the posterior end of the rostral hindbrain, as shown in Figure 4B (see “Methods”).

Following [30] we used the miRNA profile function *α*_*m*_(*x*) = *p*_1_(tanh((*l* − *x*)/*p*_2_) + *p*_3_) and in order to analyze the sharpening effect of this profile we estimated the parameters *p* = (*p*_1_,*p*_2_,*p*_3_) and *l* (the resulting profile is shown in Figure 3E). We obtained a best-fit profile according to the grayscale profile (Figure 4B red profile). The profile is in accordance with the modeling assumptions and we assumed that the expression pattern of the IsO genes is already stably established at E10.5 and this particular miRNA profile is not established before E12.5. This is evidence that the miRNA regulation of *Wnt1* in the model is not a regulatory interaction necessary to establish the pattern, but rather acts as a fine tuning mechanism to reduce the range of cells which have an intermediate *Wnt1* expression. To verify this effect the model was simulated using the E10.5 expression pattern as initial condition and the estimated profile for miRNA production. We used scenario ii) for the simulation (see Figure 4C and D), because scenario i) mostly affects the repression of *Wnt1* translation and we only have evidence for degradation with the performed experiments. We also neglect iii) as we had no experimental evidence for a diffusion of *miR-709* in the neural tube. In the simulations, a clearly visible sharpening effect could be observed, especially if we increase the *Fgf8* diffusion coefficient (see Figure 4C and D).

In contrast to *miR-709*, did not show a refined and graded expression around the ventral MHB in the E12.5 mouse embryo (see Additional file 3: Figure S3). These results indicated that only *miR-709* is expressed within the MHR and around the MHB in a pattern as predicted by the model and consistent with a possible regulatory function of *miR-709* on *Wnt1* expression at the MHB.

To determine whether *miR-709* and *miR-705* can indeed regulate the expression of *Wnt1* in a cellular context, we used the so-called luciferase “sensor assays”. In this experimental setup, a fragment of the *Wnt1 3’UTR* containing the predicted *miR-709* and *miR-705* binding sites (BS) was cloned at the 3’ end of a sequence encoding the Firefly luciferase protein. This constitutes the so-called “sensor vector”. The luciferase protein transfected with the sensor vector are indirectly measured by a bioluminescence reaction resulting in the emission of light, and the intensity of the emitted light is directly proportional to the luciferase protein concentration in the cells. Co-transduction of the cells with the *Wnt1 3’UTR* sensor vector and *miR-709* or *miR-705* should result in a reduction of luciferase protein levels, relative to an “empty” control vector (that does not contain any *Wnt1 3’UTR* sequences), if and only if these miRNAs bind to their target sites within the *Wnt1 3’UTR* and post-transcriptionally inhibit the expression of luciferase protein in these cells. Indeed, co-transduction of HEK-293T cells with the *Wnt1 3’UTR* sensor vector and *miR-709* resulted in an approx. 23% reduction of luciferase bioluminiscence in these cells (see Additional file 3: Figure S2), whereas co-transduction of HEK-293T cells with the *Wnt1 3’UTR* sensor vector and *miR-705* had no significant effect (see Additional file 3: Figure S3). This result indicated that *miR-709*, but not *miR-705*, indeed targets the *Wnt13’UTR*. Next, we determined whether the post-transcriptional regulation of *Wnt1* expression by *miR-709* is indeed mediated by the predicted *miR-709* BS within the *Wnt1 3’UTR*. Therefore, the predicted BS sequences were changed such that they could not be bound anymore by *miR-709* (see Additional file 4: Table S1) and consequently the expression levels of luciferase protein should not be affected relative to the control (“empty”) sensor vector. Mutagenesis of the two predicted *miR-709* BS within the *Wnt1 3’UTR* in fact abolished the negative regulation of luciferase expression from the sensor vector by *miR-709*. This result indicated that the negative post-transcriptional regulation of the *Wnt1* mRNA by *miR-709* is in fact mediated by the two predicted *miR-709* BS within the *Wnt1 3’UTR*.

## Discussion

### Two-scale models allow for the simulation and analysis of complex patterning processes including discontinuities

As many biological processes involve different spatial scales, multi-scale models become increasingly important in computational biology. However, the methods available to simulate and analyze these models rigorously, are still limited. In this work, we considered the two-scale processes responsible for the formation of the gene expression pattern constituting the IsO. This process involves highly nonlinear dynamics given by the gene regulatory networks in the single cells, as well as the diffusion of morphogens on the tissue scale. While the dynamics of the tissue scale are defined by a typical morphogen based patterning process, which has been extensively studied [2], the single cell system considered here is capable of generating discontinuous expression profiles. Due to numerical diffusion, common numerical methods fail to converge for this class of models [10]. To circumvent this problem, we used an algorithmic scheme which exploits the structure of the model, namely the two-scale nature. It merely uses finite differences in the spatial coordinates, which have been successfully applied to reaction-diffusion type models [38], and stiff, adaptive solvers for the time integration. Beyond standard patterning systems, based on mass-action kinetics, the method applied can solve systems with highly nonlinear interaction terms. As Lipschitz continuity and boundedness of the activation function is the only prerequisite for the existence and the boundedness of solution, the results are widely applicable.

The two-scale modeling approach and the tailored simulation scheme are used to analyze the dynamics of MHB formation and the corresponding steady state expression pattern. This problem has been approached previously, however, direct numerical integration using common PDE solvers merely allowed for the study of the short-term response [10]. To study the systems in the stationary limit, spectral methods were employed [12]. For the model published by Wittmann et al. [10], the spectral method determined four steady states, two of which were biologically plausible. However, the spectral methods provided only an approximation of the steady state distributions, as they were based on a reduced model and the discontinuities had to be predefined. This approach indirectly constrained the state space of the model and the simulation-based stability analysis we carried out revealed that one of these steady states was unstable, while the other one was stable and corresponds to the steady state shown in Figure 2B in the left lower panel, with diffusion coefficients set to 0.01. However, the steady state depicted in Figure 2B in the right lower panel was not approximated by spectral methods, illustrating their limitations.

### Spatio-temporal model of MHB formation predicts post-transcriptional miRNA regulation

To determine the plausibility of the existing models, the computed steady state profiles were compared to the experimentally observed expression profiles. While simulation results and experimental observations agree qualitatively, the model fails to describe the *Wnt1* profile adequately. The obtained expression domain had a smooth gradient like form which contrasts the experimental findings where the expression domain is a sharply delimited ring around the MHB [8].

The sharpening of the *Wnt1* profile could be caused by different mechanisms, ranging from additional transcriptional regulation to post-transcriptional regulation. In this work, we focused on miRNA-mediated regulation as miRNA have proven to be essential in embryonic patterning processes including morphogens, e.g. in zebrafish [39,40]. Furthermore, we already established the importance of miRNAs in general brain development (unpublished data). However, a role of miRNAs in the formation of the MHB and in the refinement of the *Wnt1* expression pattern at this boundary has not been reported so far.

Using our model, we could verify that different miRNA-mediated regulation mechanisms can cause a sharpening of the *Wnt1* expression domain at the MHB. This sharpening can be induced by different mechanisms, for which we provided a generalized mathematical model. In contrast to existing models for post-transcriptional miRNA regulation we thereby also considered the partial recycling of the miRNA and did not assume that the mRNA-miRNA complex is degraded instantaneously, which is biologically often not plausible. Given this theoretical result, we performed a problem-tailored miRNA target prediction. Two candidate miRNAs, *miR-705* and *miR-709*, were identified and analyzed experimentally. The *in situ* hybridization (detection) experiments showed a promising qualitative expression profile for *miR-709*, which is in line with the predictions made by the model. However, it did not yield insight into the detailed interactions or the necessity of *miR-709* for MHB development, which would require the establishment of a knock-out, knock-down and/or over-expression mouse model for *miR-709*.

Beyond the complete verification of the miRNA-based regulation of *Wnt1* expression *in vivo*, another question that arises about the mechanism behind the formation of the observed gradient of *miR-709* expression across the MHB. Possible mechanisms include the regulation of *miR-709* expression by external factors or by a direct feedback regulated by *Fgf8* or other factors involved in the formation of the MHB. The latter could give rise to positive feedback and further sharpening not yet considered in the model.

Apart from a post-transcriptional regulation of *Wnt1* mRNA expression by miRNAs, additional regulatory mechanisms might also influence the formation of sharp *Wnt1* expression boundaries at the MHB. An example is the transcription factor *Lmx1b*, which is known to maintain *Wnt1* expression [41,42] at the MHB at E10.5. As this factor is expressed only in a ring around the MHB, it might contribute to the sharpening of the domain. Other signaling and additional cell-cell communication processes can also not be ruled out. Additional studies are necessary to unravel or exclude further mechanisms involved in the fine tuning of the IsO gene expression profiles during mouse (vertebrate) embryonic development.

## Conclusion

To understand complex patterning processes quantitatively, spatial-temporal modeling has been proven to be essential, for example in Drosophila. In this contribution, we illustrated how even a model using only a semi-quantitative description of the gene regulatory network acting on a tissue scale can help to guide the discovery of novel regulation mechanisms, in our case gene expression boundary sharpening induced by post-transcriptional feedback mechanisms. As spatially resolved data increases quickly, methods employing also spatial information will become more and more important.

## Methods

### Numerical integration

A characteristic of semi-quantitative, macroscopic models of two-scale processes is the development of spatially discontinuous functions for the cell specific transcription factors (see position marked with dashed circles in Figure 2). A standard heat equation solver or spectral methods cannot solve this models without prior knowledge of the discontinuity’s position. The numerical integration method must solve the PDE as well as the increasingly discontinuous solutions for the ODEs without mollifying. We used a semi-discretization in space so if △_*x*_ denotes a discretized Laplace operator we obtained an initial value problem for ODEs given by

$$\frac{\text{du}}{\text{dt}}(t,x_{i})=f(u(t,x_{i}))+D\frac{\partial^{2}}{\partial x^{2}}u(t,x_{i}),\;u(0,x_{i})=u_{0}(x_{i}),$$

for each grid point *x*_*i*_, *i* = 1,…*N*. As all solutions are in $C([0,T],L^{2}([-L,L],R^{8}))$ we needed a *L*^2^ stable spatial discretization and we chose finite differences. We took into account that with *h*→0, where *h* is the grid width, the generated ODEs became increasingly stiff. The finite differences method yields a Jacobian matrix with a special sparsity pattern. The matrix is non zero only on the eight subdiagonals and the eight superdiagonals, which we use to enhance the performance of the ODE solver. The algorithm is implemented for MATLAB R2012a and can be found in Additional file 5: Data S1.

### Steady state approximation and stability analysis

We determined the steady states by simulating the model with the parameters given in the results section from different initial conditions until the calculated state of the system changed less than machine accuracy between two time steps. This was repeated for many different initial conditions, space fillingly sampled, to find as many steady states as possible with a numerical simulation.

In a second step, we identified the stability of the reached state. Therefore, we added uniform distributed noise, ϵ∼U([−0.001;0.001]), to the calculated value for each component under the constraint that *u*_*i*_(*t*,*x*) + *ϵ*≥0 for *i* = 1,…,6 and *v*_*j*_(*t*,*x*) + *ϵ*≥0 for *j* = 1,2. We used the obtained value as the initial condition for the approximation of the steady state. If the unperturbed state was reached again we concluded that the state was stable.

### Experimental animals

Outbred CD-1 mice were purchased from Charles River (Kisslegg, Germany) and kept under a 12-12 light-dark cycle under standard conditions. Mice had ad libitum access to food and water. Noon on the day of vaginal plug detection was designated as embryonic day (E) 0.5. Embryos were staged according to Theiler [43].

### miRNA prediction

To improve the robustness of the predicted miRNAs that target the *Wnt1* mRNA, data sets of five most commonly used miRNA prediction tools were used in combination. A miRNA target was considered as a candidate if the miRNA target interaction was predicted by at least three out of five miRNA target prediction tools. For the miRNA target prediction, we used the following publicly available tools: TargetScan [44], PicTar [45], miRNAMAP (miranda) [46], TargetSpy [47] and miRBase (DIANA) [48].

### Radioactive *in situ* hybridization (ISH) and probe labeling

Timed-pregnant mice were killed by *CO*_2_ asphyxiation. Uterine horns were removed and kept in cold phosphate buffered saline (PBS) before dissection of the embryos. Embryos were fixed in 4% paraformaldehyde (PFA) (Sigma, Germany) in PBS overnight, dehydrated in an ascending ethanol series, cleared in xylene, embedded in paraffin, and sectioned on a microtome (Microm, Germany) at 8 *μm* thickness. Radioactive locked nucleic acid (LNA)-based ISH using unlabeled, LNA-modified *mmu-miR-709* (Exiqon, Denmark, Cat No 39324-00) and *mmu-miR-705* (Exiqon, Cat No 39320-00) detection probes were performed using an ISH protocol as described in [33] with minor modifications: the proteinase K treatment was omitted, pre-hybridization and hybridization of the labeled probes was done in an *in situ* Hybridization Buffer (Ambion, USA, Cat No B8807G) at 53°C, and post-hybridization washes were done sequentially in 1xSSC, 0.2xSSC and 0.1xSSC at 53° C. Sections were counterstained with Cresyl Violet (0.5%, Sigma) according to standard procedures after exposure for 1–3 weeks. Images were taken with an Axioplan2 microscope using bright- and darkfield optics, AxioCam MRc camera and Axiovision 4.6 software (Zeiss, Germany), and processed with Adobe Photoshop CS5 software (Adobe Systems Inc., USA). The LNA-modified *mmu-miR-709* and *mmu-miR-705* detection probes were labeled with [ *α*^35^*S*]-dATP (GE Healthcare, USA), using the Terminal Transferase Labeling Kit (Roche, Germany) according to the manufacturer’s instructions, with minor modifications: a 1:50 dilution (0.5 *μM*) of the unlabeled LNA-modified detection probe, 1 *mCi*/*mlα*^35^*S*-dATP and no UTP were used in the reaction mixture.

### Calculation of grayscale profile and profile fitting

We defined a 300 pixel long and 15 pixel wide region from the approximate anterior end the midbrain to the approximate posterior end of the rostral hindbrain (both marked by dashed red lines in Figure 4A) on a darkfield picture taken from a sagittal sections of an E12.5 wild-type embryo hybridized with the radioactive *mmu-miR-709* detection probe). Using the software ImageJ (NIH, USA), we calculated the gray value in this picture at each pixel within the rectangular area in Figure 4A and averaged the values along the width of the rectangular. The gray value profile obtained was normalized against the mean gray value intensity in the two light blue squares/boxes shown in Figure 4A. We estimated the parameters *p* = (*p*_1_,*p*_2_,*p*_4_) and *l* of the profile function *α*_*m*_(*x*) = *p*_1_(tanh((*l* − *x*)/*p*_2_) + *p*_4_) (suggested in [30]). Therefore, we minimized the quadratic distance $\overset{300}{\underset{i=1}{\sum }}{\left(D(x_{i})-\alpha_{m}(x_{i},p,l))\right)}^{2}$, with pixels *x*_*i*_ and gray value *D*(*x*_*i*_), using the minimization method fminsearch implemented in MATLAB R2012a. The optimal parameters are *p*_1_ = 0.3062, *l* = 0.451, *p*_2_ = 0.2, *p*_3_ = 0.064 and *p*_4_ = 2.2868 and the least squares fit of *α*_*m*_(*x*) to the gray value curve *D*(*x*_*i*_) is shown in in Figure 3E.

### Luciferase sensor assays

A 857 bp fragment of the *Wnt1 3’UTR* (Entrez Gene Acc. No. NM_021279, basepairs 1496-2352) was amplified from the E12.5 mouse embryo head cDNA using the primer pair shown in Additional file 4: Table S1. This *Wnt1 3’UTR* fragment contains two putative BS each for *mmu-miR-709* and for *mmu-miR-705* as predicted by miRBase (microCosm). This fragment was subsequently subcloned into the *Xba*I site located downstream of the firefly luciferase stop codon in the pGL3 Promoter vector (Promega, USA). Site-directed mutagenesis of the predicted *mmu-miR-709* seed sequences within the 857 bp *Wnt1 3’UTR* fragment was done using the Quick Change Multi-Site Directed Mutagenesis Kit (Stratagene, USA) according to the manufacturer’s instructions. Mutagenic primers used are shown in Table S4. HEK293T (1×10^5^ cells/well) were plated in a 24-well plate and co-transfected 24 hours later with 300 ng of*Wnt1 3’UTR-WT* or *Wnt1 3’UTR-MUT* sensor vector, 30 ng of renilla luciferase vector, and *mmu-miR-709* (Ambion, Cat No PM11496) or *mmu-miR-705* (Ambion, Cat No PM11392) precursor miRNA as indicated in the figures, using Lipofectamine 2000 (Invitrogen) according to the manufacturer’s instructions. Luciferase activity was measured 48 hours after transfection using the Dual-Luciferase Reporter Assay System (Promega). The firefly luciferase values were normalized against the renilla luciferase values as internal transfection control. As we also observed a down-regulation of luciferase activity after co-transfection of the precursor miRNA and the pGL3 Promoter vector (without any 3’UTR cloned into it) in some instances, which we considered to be “off-target” effects of the corresponding miRNA, we always used the co-transfection of pGL3 Promoter vector without 3’UTR (“empty vector”) and the corresponding miRNA as the control in our experiments, and this value was set as one. Transfections were done in triplicate and all data derive from three independent experiments.

### Ethics statement

Animal treatment was conducted under federal guidelines for the use and care of laboratory animals and was approved by the HMGU Institutional Animal Care and Use Committee.

## Competing interests

The authors declare that they have no competing interests.

## Authors’ contributions

SH wrote the paper, conceived and designed methodology and performed computational experiments. YN wrote paper, conceived and designed experiments and analyzed the data. JH and DW conceived and designed methodology and helped draft the manuscript. DL and DT performed the miRNA target scan. WW coordinated the experimental work. NP coordinated experimental work, conceived and designed experiments and helped draft the manuscript. FJT conceived and designed the methodology and coordinated theoretical work. All authors read and approved the final manuscript.

## Supplementary Material

Additional file 1 **Figure S1.** Work flow of miRNA database search. (A) The target prediction work flow. (B) The network of target genes (orange) used for the prediction and the predicted miRNAs (green). Click here for file

Additional file 2 **Figure S2.***mmu-miR-709* is expressed in the MHR close to the MHB of the developing mouse embryo and targets the *Wnt1 3’UTR* in vitro.(A-D) Representative images of sagittal sections through an E10.5 (A,C) and E12.5 (B,D) CD-1 mouse embryo, hybridized with a radioactive *mmu-miR-709* LNA oligonucleotide probe. (C,D) are enlarged dark-field views of the boxed areas in (A,B). At E10.5, *miR-709* is expressed strongly in the anterior neural tube including hindbrain, midbrain and part of the forebrain (diencephalon), but sparing the major part of the forebrain. (A,C). At E12.5, *miR-709* is strongly expressed in the dorsal telencephalon (cortex), diencephalon (thalamus), anterior midbrain and caudal hindbrain (rhombomere 1), and apparently weaker in the rostral rhombomere 1 and around the ventral MHB (B,D). (E) Schematic drawing of the *Wnt1 3’UTR* sensor vector showing the approximate position of the two *miR-709* seed sequences (binding sites) predicted by miRBase (microCosm) within the *Wnt1 3’UTR* and of the mutated *Wnt1 3’UTR* sensor vector (mutant, Mt). (F) Sequence of the two *mmu-miR-709* binding sites in the *Wnt1 3’UTR*, with the conserved 7 nt seed sequence highlighted in blue. (G) Luciferase sensor assays after co-transfection of 30 nM *mmu-miR-709* precursor miRNA and a sensor vector that (a) does not contain any 3’UTR (“empty vector”) or (b) a sensor vector containing the *Wnt1 3’UTR* at the 3’ end of the Luciferase CDS show that *miR-709* down-regulates *Wnt1 3’UTR*-mediated Luciferase expression by approx. 23% (*Wnt1 3’UTR + miR-709*:0.771 ± 0.037, mean ± sd) relative to the empty vector control. Site-directed mutagenesis of both seed sequences within the *Wnt1 3’UTR* sensor vector (*Mt-Wnt1 3’UTR*) abolished this negative regulation (*Mt-Wnt1 3’UTR + miR-709*:0.93 ± 0.067, mean ± sd) (single asterisk, *p*<0.05; double asterisk, *p*<0.01; student’s-T-test). Abbreviations: Di, diencephalon; Fb, forebrain; Hb, hindbrain; Mb, midbrain; Mes, mesencephalon; Met, metencephalon; MHB, mid-hindbrain boundary; r1, rhombomere 1; Tel, telencephalon Click here for file

Additional file 3 **Figure S3.***mmu-miR-705* is expressed in the MHR close to the MHB of the developing mouse embryo but does not target the *Wnt1 3’UTR* in vitro. (A-D) Representative images of sagittal sections through an E10.5 (A,C) and E12.5 (B,D) CD-1 mouse embryo, hybridized with a radioactive *mmu-miR-705* LNA oligonucleotide probe. (C,D) are enlarged dark-field views of the boxed areas in the bright-field overviews shown in (A,B). (E) Schematic drawing of the *Wnt1 3’UTR* sensor vector showing the approximate position of the two *miR-705* seed sequences (binding sites) predicted by miRBase (microCosm). (F) Sequence of the two *mmu-miR-705* binding sites in the *Wnt1 3’UTR*. (G) Luciferase sensor assays after co-transfection of *mmu-miR-705* precursor miRNA and a sensor vector that (a) does not contain any 3’UTR (“empty vector”) or b a sensor vector containing the *Wnt1 3’UTR* at the 3’ end of the Luciferase CDS show that *miR-705* does not significantly down-regulate *Wnt1 3’UTR*-mediated Luciferase expression. Abbreviations: Di, diencephalon; Fb, forebrain; Hb, hindbrain; Mb, midbrain; Mes, mesencephalon; Met, metencephalon; MHB, mid-hindbrain boundary; r1, rhombomere 1; Tel, telencephalon. Click here for file

Additional file 4 **Table S1.** miRNA binding sites. Click here for file

Additional file 5 **Data S1.** MATLAB files for simulation. Click here for file
