# Supplementary figures and images for: Sharpening of expression domains induced by transcription and microRNA regulation within a spatio-temporal model of mid-hindbrain boundary formation
Source: BMC Syst Biol. 2013 Jun 25;7:48. doi: 10.1186/1752-0509-7-48 (PMC4103684; doi:10.1186/1752-0509-7-48)

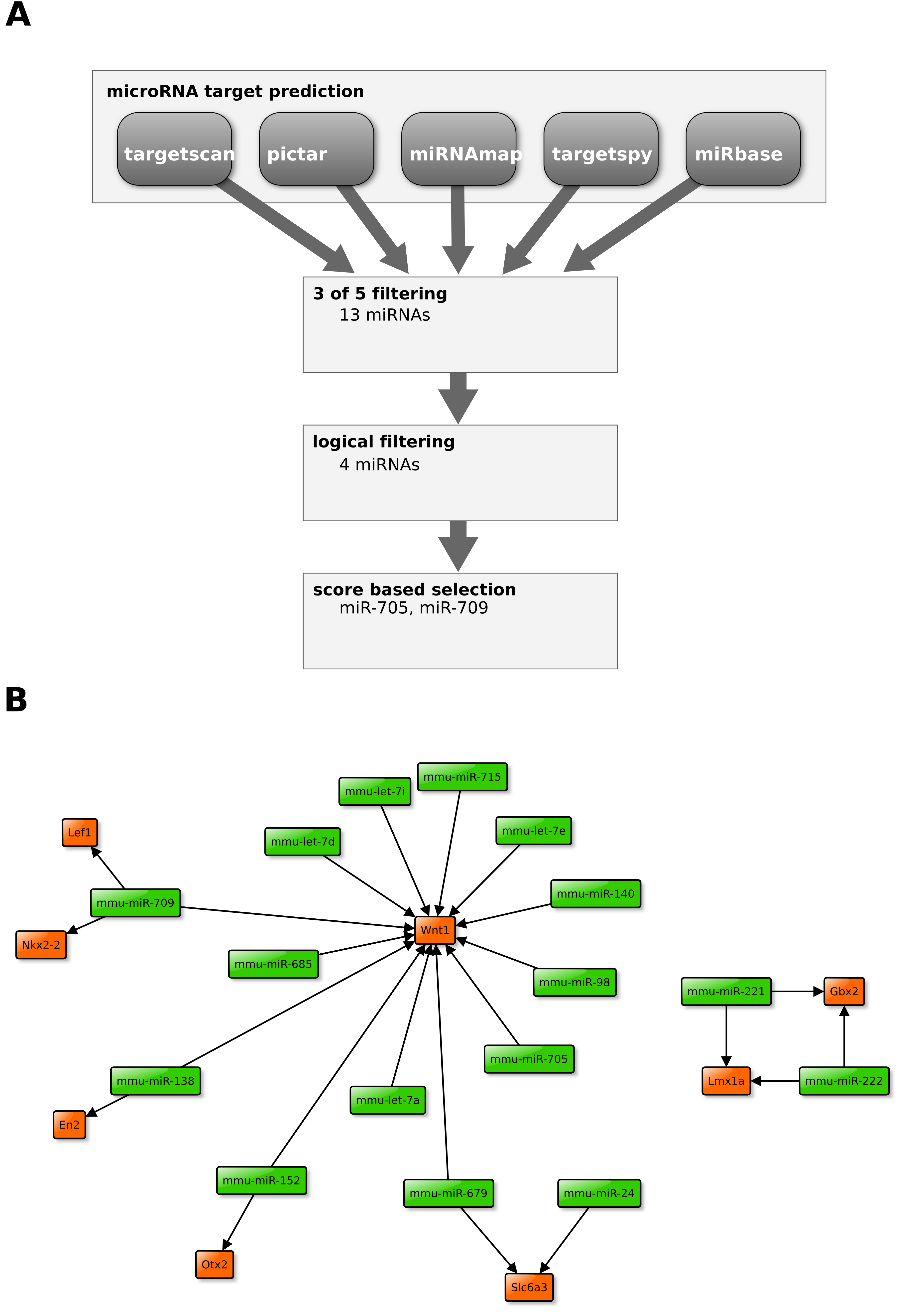

Supplement: Additional file 1 — Figure S1. Work flow of miRNA database search. (A) The target prediction work flow. (B) The network of target genes (orange) used for the prediction and the predicted miRNAs (green). [file 1752-0509-7-48-S1.tiff]

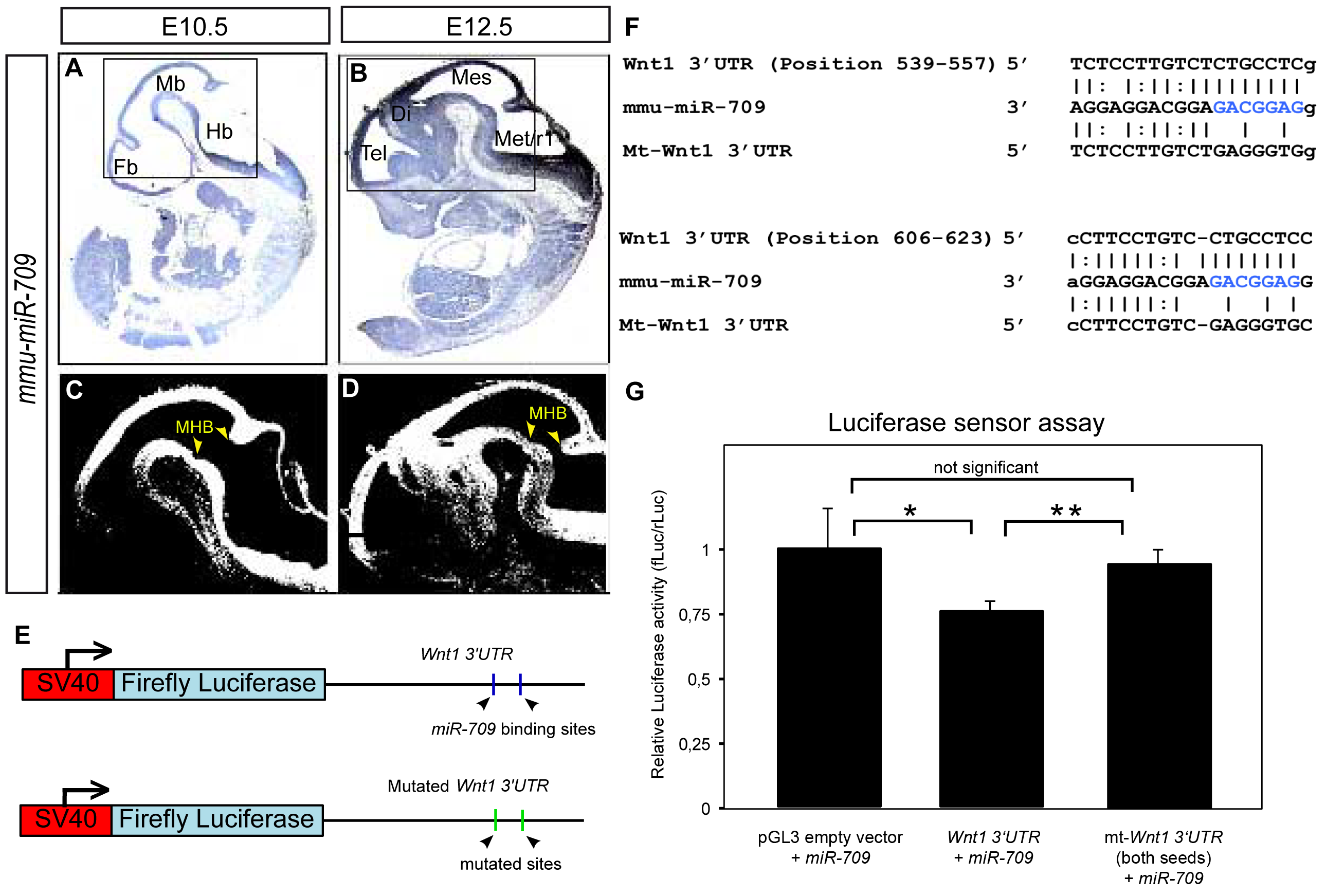

Supplement: Additional file 2 — Figure S2.mmu-miR-709 is expressed in the MHR close to the MHB of the developing mouse embryo and targets the Wnt1 3’UTR in vitro.(A-D) Representative images of sagittal sections through an E10.5 (A,C) and E12.5 (B,D) CD-1 mouse embryo, hybridized with a radioactive mmu-miR-709 LNA oligonucleotide probe. (C,D) are enlarged dark-field views of the boxed areas in (A,B). At E10.5, miR-709 is expressed strongly in the anterior neural tube including hindbrain, midbrain and part of the forebrain (diencephalon), but sparing the major part of the forebrain. (A,C). At E12.5, miR-709 is strongly expressed in the dorsal telencephalon (cortex), diencephalon (thalamus), anterior midbrain and caudal hindbrain (rhombomere 1), and apparently weaker in the rostral rhombomere 1 and around the ventral MHB (B,D). (E) Schematic drawing of the Wnt1 3’UTR sensor vector showing the approximate position of the two miR-709 seed sequences (binding sites) predicted by miRBase (microCosm) within the Wnt1 3’UTR and of the mutated Wnt1 3’UTR sensor vector (mutant, Mt). (F) Sequence of the two mmu-miR-709 binding sites in the Wnt1 3’UTR, with the conserved 7 nt seed sequence highlighted in blue. (G) Luciferase sensor assays after co-transfection of 30 nM mmu-miR-709 precursor miRNA and a sensor vector that (a) does not contain any 3’UTR (“empty vector”) or (b) a sensor vector containing the Wnt1 3’UTR at the 3’ end of the Luciferase CDS show that miR-709 down-regulates Wnt1 3’UTR-mediated Luciferase expression by approx. 23% (Wnt1 3’UTR + miR-709:0.771 ± 0.037, mean ± sd) relative to the empty vector control. Site-directed mutagenesis of both seed sequences within the Wnt1 3’UTR sensor vector (Mt-Wnt1 3’UTR) abolished this negative regulation (Mt-Wnt1 3’UTR + miR-709:0.93 ± 0.067, mean ± sd) (single asterisk, p<0.05; double asterisk, p<0.01; student’s-T-test). Abbreviations: Di, diencephalon; Fb, forebrain; Hb, hindbrain; Mb, midbrain; Mes, mesencephalon; Met, metencephalon; MHB, mid-hindbrain bou [file 1752-0509-7-48-S2.tiff]

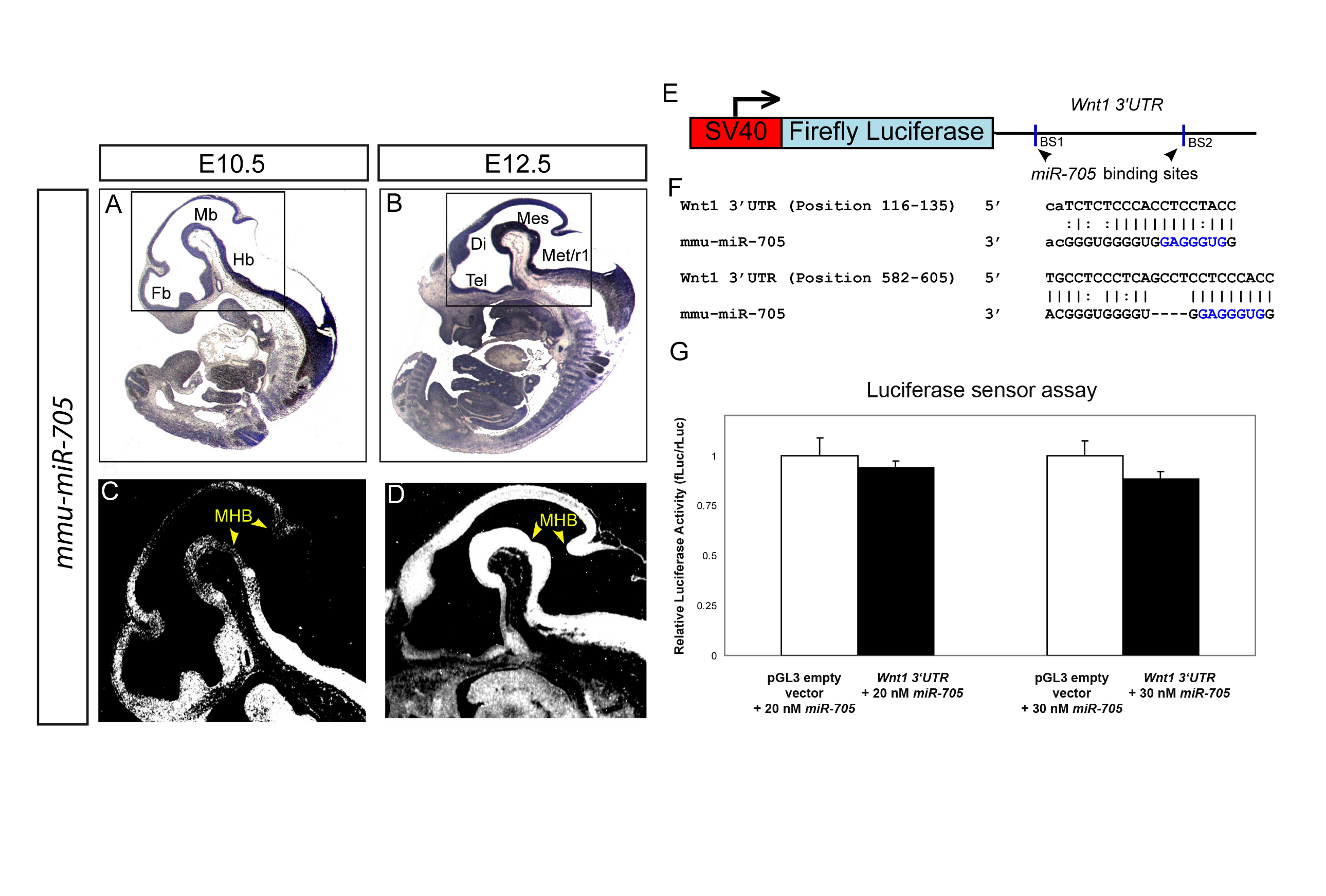

Supplement: Additional file 3 — Figure S3.mmu-miR-705 is expressed in the MHR close to the MHB of the developing mouse embryo but does not target the Wnt1 3’UTR in vitro. (A-D) Representative images of sagittal sections through an E10.5 (A,C) and E12.5 (B,D) CD-1 mouse embryo, hybridized with a radioactive mmu-miR-705 LNA oligonucleotide probe. (C,D) are enlarged dark-field views of the boxed areas in the bright-field overviews shown in (A,B). (E) Schematic drawing of the Wnt1 3’UTR sensor vector showing the approximate position of the two miR-705 seed sequences (binding sites) predicted by miRBase (microCosm). (F) Sequence of the two mmu-miR-705 binding sites in the Wnt1 3’UTR. (G) Luciferase sensor assays after co-transfection of mmu-miR-705 precursor miRNA and a sensor vector that (a) does not contain any 3’UTR (“empty vector”) or b a sensor vector containing the Wnt1 3’UTR at the 3’ end of the Luciferase CDS show that miR-705 does not significantly down-regulate Wnt1 3’UTR-mediated Luciferase expression. Abbreviations: Di, diencephalon; Fb, forebrain; Hb, hindbrain; Mb, midbrain; Mes, mesencephalon; Met, metencephalon; MHB, mid-hindbrain boundary; r1, rhombomere 1; Tel, telencephalon. [file 1752-0509-7-48-S3.tiff]
