# Supplementary material for: Sharpening of expression domains induced by transcription and microRNA regulation within a spatio-temporal model of mid-hindbrain boundary formation
Source: BMC Syst Biol. 2013 Jun 25;7:48. doi: 10.1186/1752-0509-7-48 (PMC4103684; doi:10.1186/1752-0509-7-48)
Supplement: Additional file 4 — Table S1. miRNA binding sites. [file 1752-0509-7-48-S4.doc]

**Supporting information Table 1**

| Name | Length (bp) | Accession No. | Primer sequence | Tm (°C) |
| --- | --- | --- | --- | --- |
| *Wnt1*  *3’ UTR* | 857 | NM_021279 | Forward primer: 5’-CTCGCTGGTCCTGATGTTTG-3’  Reverse Primer: 5’-ACCAATGTAGTATCTTCCTCTGAGG-3’ | 58 |
| *Wnt1_Mut1*  *3’ UTR mutant BS1** | 857 |  | Forward: 5'-CAAAACCCTACATTCTCCTTGTCT**GA**G**GG**T**G**GGAGCCATTGAACAGC-3'  Reverse: 5'-GCTGTTCAATGGCTCC**C**A**CC**C**TC**AGACAAGGAGAATGTAGGGTTTTG-3' | 55  60 |
| *Wnt1_Mut2*  *3’ UTR mutant BS2** | 857 |  | Forward: 5'- CCTCCTCCCACCCCTTCCTGTC**GA**G**GG**T**G**CTCATCACTGTGTAA-3'  Reverse: 5'- TTACACAGTGATGAG**C**A**CC**C**TC**GACAGGAAGGGGTGGGAGGAGG-3' | 55  60 |

* BS = binding site
